# Nitro Derivatives of Naturally Occurring ***β***-Asarone and Their Anticancer Activity

**DOI:** 10.1155/2014/835485

**Published:** 2014-10-01

**Authors:** Suvarna Shenvi, Latha Diwakar, G. Chandrasekara Reddy

**Affiliations:** ^1^Chemical Sciences Division, Vittal Mallya Scientific Research Foundation, BTM II Stage, Bangalore 560076, India; ^2^Department of Biological Sciences, Vittal Mallya Scientific Research Foundation, BTM II Stage, Bangalore 560076, India

## Abstract

*β*-Asarone (2, 4, 5-trimethoxy-(*Z*)-1-propenylbenzene) was obtained from *Acorus calamus.* Nitration of *β*-asarone with AgNO_2_/I_2_ in ether yielded 1-(2, 4, 5-trimethoxy phenyl)-2-nitropropene (**1**) but with NaNO_2_/I_2_ in ethylene glycol obtained 1-(2, 4, 5-trimethoxy phenyl)-1-nitropropene (**2**). Compound **2** was prepared for the first time and characterized using IR, ^1^H-NMR, ^13^C-NMR, and GC-MS spectra and it was converted into 1-(2, 4, 5-trimethoxy) phenyl-1-propanone (**3**) using modified Nef reaction. Based on 1D NOESY experiments, compounds **1** and **2** have been assigned *E* configuration. Compounds **1** and **2** were subjected to cytotoxic activity using five human cancer cell lines, namely, MCF-7, SW-982, HeLa, PC-3, and IMR-32 by MTT assay. Except in breast cancer line (MCF-7) compound **2** exhibited five- to tenfold increase in activity compared to *β*-asarone and twofold increase over compound **1**.

## 1. Introduction

*Acorus calamus* (Acoraceae) also known as sweet flag in Indian traditional medicine is generally used for treatment of cough, fever, bronchitis, inflammation, depression, tumors, haemorrhoids, skin diseases, insomnia, hysteria, epilepsy, and loss of memory [1, 2]. While *β*-asarone (2, 4, 5-trimethoxy-(*Z*)-1-propenylbenzene) was the main constituent (70 to 90%) of rhizomes of* Acorus calamus* [3], α-asarone (2, 4, 5-trimethoxy-(*E*)-1-propenylbenzene) was isolated as a minor component (8 to 14%) from the rhizomes of related species* Acorus gramineus* [4]. Comparative study of genotoxicity and cytotoxicity of *β*-asarone and α-asarone was investigated and found that α-asarone was more toxic in the HepG2 cell system [5–7].

In continuation of our research on *β*-asarone, we carried out different chemical conversions to get pharmacologically active compounds [8, 9]. Herein we report the preparation of nitro derivatives of *β*-asarone and their biological activity. Nitro group is an important functional group because it can be easily converted into many functional groups [10–12]. Psychoactive drugs, namely, amphetamines, are generally prepared by reduction of *β*-methyl-*β*-nitrostyrenes [13–16].

Generally nitration of alkenes and substituted styrenes was carried out by metal nitrites using NaNO_2_/AgNO_2_ with iodine, NaNO_2_/H_2_SO_4_ in ether (Bruckner's method), Cu (II)tetrafluoroborate with NaNO_2_, alkyl halide and metal nitrite (Victor-Meyer reaction), and HgCl_2_-NaNO_2_ [17–23]. Formation of nitryl iodide was first reported by Birchenbachin 1932 from AgNO_2_/I_2_ [24]. Later on AgNO_2_ was replaced with less expensive NaNO_2_/H_2_O/I_2_/EtOAc/ethylene glycol or KNO_2_/18-crown-6/I_2_/THF [25–27].

## 2. Results and Discussion

### 2.1. Chemistry

**Scheme 1:**
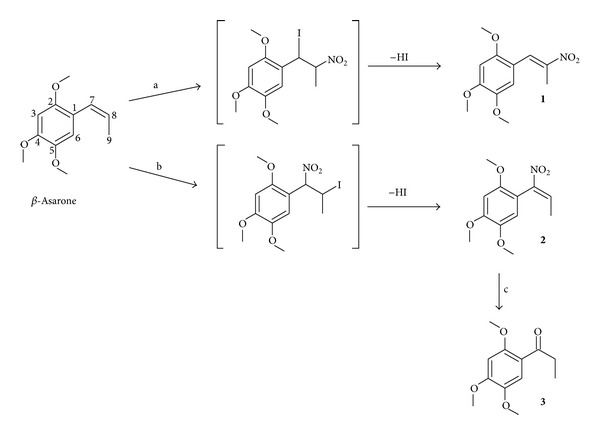
Synthesis of 1-(2, 4, 5-trimethoxy phenyl)-2-nitropropene** (1)**, 1-(2, 4, 5-trimethoxy phenyl)-1-nitropropene (**2**), and 1-(2, 4, 5-trimethoxy)phenyl-1-propanone (**3**).* Reagents and Conditions*. (a) AgNO_2_, I_2_, ether, room temperature 30 h; (b) NaNO_2_, I_2_, ethylene glycol, room temperature 48 h; (c) NaBH_4_, MeOH, room temperature 30 min.

Naturally occurring *β*-asarone when subjected to nitration with nitryl iodide (NaNO_2_/I_2_/ethylene glycol) obtained 1-(2, 4, 5-trimethoxy phenyl)-1-nitropropene (**2**) (Scheme 1) as yellow crystals. It showed a molecular ion peak at 253 (M^+.^) corresponding to the molecular formula C_12_H_15_NO_5_. In ^1^H-NMR spectrum methyl group appeared as a doublet at *δ*_*H*_ **1.80 (*J* = 7.6 Hz) with vinylic proton appearing as a quartet at*δ*_*H*_ **7.40 (*J* = 14.6 and 7.6 Hz) indicating that vinylic proton is adjacent to methyl group. When the methyl peak in PMR at *δ* **1.80 was irradiated, enhancement of peaks at *δ* **6.67 and *δ* **7.40 corresponding to H-6 and vinylic proton was observed indicating that compound** 2** is indeed 1-(2, 4, 5-trimethoxy phenyl)-(*E*)-1-nitropropene. The formation of compound** 2 **is quite unique and there was no report of formation of this earlier. Further proof that compound** 2 **is 1-nitropropenyl derivative has come from the fact that when it was subjected to modified Nef reaction using sodium borohydride (wherein α,*β*-unsaturated nitroalkenes yield corresponding ketones by hydrolysis of the corresponding nitronates [28, 29], it gave 1-(2, 4, 5-trimethoxy)phenyl-1-propanone (**3**, isoacoromone)) [3]. Sy and By reported that nitration of substituted styrenes with nitryl iodide regioselectively yielded *β*-nitrostyrenes (2-nitropropenyl derivatives) and not α-nitrostyrenes (1-nitropropenyl derivative) [24]. We have also prepared 1-(2, 4, 5-trimethoxy phenyl)-2-nitropropene (**1**) by the nitration of *β*-asarone with AgNO_2_/I_2_ in ether and characterized it by recording its PMR spectrum. When the methyl peak in PMR at *δ* **2.42 of compound** 1** was irradiated, no enhancement of any other peaks was observed. The formation of compound** 1** was confirmed by synthesizing the molecule through the condensation of 2, 4, 5-trimethoxy benzaldehyde with nitroethane [30, 31]. It is clear from these experiments that formation of nitro derivatives of *β*-asarone depends on the solvent used during nitration.

### 2.2. Biological Activity

Compounds** 1 **and** 2** were screened for their anticancer activity against five human cancer cell lines by MTT assay (Table 1) with the naturally occurring *β*-asarone and camptothecin taken as standards. Except in breast cancer cell line (MCF-7), compound** 2 **exhibited five- to tenfold increase in activity compared to *β*-asarone and twofold increase over compound** 1**.

#### 2.2.1. Anticancer Assay

Cell lines were maintained in Dulbecco's Modified Eagle's Medium (Sigma-Aldrich Inc., USA) supplemented with 10% fetal bovine serum (Gibco BRL., USA) in a CO_2_ incubator at 37°C. The cytotoxicity of the compounds was measured by MTT assay [32]. Five different kinds of human cancer cell lines, namely, HeLa (cervical), MCF-7 (breast), SW-982 (synovial), PC-3 (prostate cancer), and IMR-32 (neuroblastoma), were plated in a 96-well plate at the density of 10,000 cells per well. After 24 h, cells were treated with various concentrations of compounds from 200 *μ*M serially diluted up to 1.56 *μ*M using camptothecin as standard. The cells were further incubated for 48 h, and 20 *μ*L of MTT (5 mg/mL stock, Sigma-Aldrich Inc., USA) was added to each well and incubated for another three hours. The purple formazan crystals formed were dissolved by adding 100 *μ*L of DMSO to each well and absorbance was read at 570 nm in a spectrophotometer [SpectraMax 340]. The cell death was calculated as follows:

(1) $$\begin{array}{c} \text{Cell death}=100-\left[\left(\frac{\text{test absorbance}}{\text{control absorbance}}\right)\times 100\right]. \end{array}$$

The cytotoxic activity of compounds was expressed as the concentration in *μ*M at which they inhibit the cell growth by 50% (IC_50_).

## 3. Experimental

### 3.1. Chemistry

All chemicals were purchased from Laboratory Reagent (LR) grade. Fresh rhizome of* Acorus calamus* was collected from marshy areas of Kunigal in Karnataka, India in 2012. Melting points were recorded on an Acro melting point apparatus using a calibrated thermometer. Thin layer chromatography (TLC) and column chromatography (CC) were performed with [TLC silica gel 60 F_254_. Merck] and silica gel (Kieselgel 60, 230–400 mesh, Merck), respectively. Chromatograms were developed using hexane-EtOAc (8 : 2, v/v). IR spectra were recorded on Thermo-Nicolet instrument in KBr discs. Mass spectra were recorded using GCMS-QP2010S (direct probe). PMR spectra and ^13^C NMR spectra were recorded in CDCl_3_ with TMS (tetramethylsilane) as an internal standard on a Bruker AG spectrometer and chemical shifts were recorded in *δ* units.

### 3.2. 3-(2, 4, 5-Trimethoxy-(*Z*)-1-propenyl benzene)

IR (KBr): 3421, 2939, 1589, 1512, 1469, 1211, 1145, 1029, 833 cm^−1^.

^1^H NMR (CDCl_3_, 200 MHz): *δ* 1.88 (3H, dd, *J* = 2.0, 7.0 Hz, CH_3_), 3.81 (3H, s, OCH_3_), 3.84 (3H, s, OCH_3_), 3.90 (3H, s, OCH_3_), 5.78 (1H, m, H-8), 6.49 (1H, m, H-7), 6.53 (1H, s, H-3), 6.84 (1H, s, H-6).

^13^C NMR (CDCl_3_, 50 MHz): *δ* 153.58, 150.58, 143.26, 126.74, 126.42, 119.32, 116.71, 97.28, 56.98 (OCH_3_), 56.53 (OCH_3_), 56.38 (OCH_3_), 14.78 (C-9).

GC-MS (*m*/*z*) = 208 [M]^+^ (25), 193 (10), 165 (15), 150 (5), 135 (12), 119 (5), 105 (5), 91 (14).

### 3.3. 1-(2, 4, 5-Trimethoxy phenyl)-2-nitropropene (**1**)

Iodine (1016 mg, 4 mmol) and AgNO_2_ (616 mg, 4 mmol) were stirred in anhydrous ether (20 mL) at room temperature under nitrogen for 45 min. *β*-Asarone (236 mg, 2 mmol) and pyridine (632 mg, 8 mmol) in ether were added and the mixture was stirred at room temperature for 30 h as per the reported procedure [24]. The dark brown liquid material obtained was chromatographed on silica and eluted with hexane/ethyl acetate to give pure product** 1** which was crystallized from methanol (1-(2, 4, 5-trimethoxy phenyl)-2-nitropropene exists in two modifications, yellow and red prisms, and depending on concentration and precipitation speed, one often gets a mixture of both species. Yellow crystal melts at 98–100°C and dark orange (red) crystal melts at 99–101°C. The red form transforms itself to the yellow form at 90°C) (238 mg, 73.2%).

IR (KBr): 2945, 1612, 1509, 1490, 1335, 1278, 1216, 1136 cm^−1^.

^1^H NMR (CDCl_3_, 400 MHz): *δ* 2.42 (3H, s, H-9), 3.85 (3H, s, OCH_3_), 3.87 (3H, s, OCH_3_), 3.94 (3H, s, OCH_3_), 6.54 (1H, s, H-3), 6.87 (1H, s, H-6), 8.30 (1H, s H-7).

^13^C NMR (CDCl_3_, 100 MHz): *δ* 153.58, 152.44, 145.58, 142.26, 128.74, 133.01, 111.64, 97.28, 56.78 (OCH_3_), 56.53 (OCH_3_), 56.05 (OCH_3_), 14.58 (CH_3_).

GC-MS (*m*/*z*) = 253 [M^+.^] (42), 207 (100), 192 (70), 177 (62), 161 (28), 149 (25), 131 (12), 121 (30), 107 (12).

### 3.4. Synthesis of **1**

To a solution of 2, 4, 5-trimethoxy benzaldehyde (7.6 g, 38.7 mmol) in nitroethane (27 g, 386 mmol) was added ammonium acetate (1.7 g, 22.0 mmol) and the reaction mixture heated to 75–80°C for 3 hrs. After completion of the reaction, nitroethane was removed under vacuum, and oily orange mass was triturated with hot methanol (3 × 50 mL). Methanol extract was concentrated and allowed to stand at room temperature. Yellow crystals (75–78% yield) were obtained having the melting point 98–100°C. 1-(2, 4, 5-Trimethoxy phenyl)-2-nitropropene obtained by nitration of *β*-asarone using AgNO_2_/I_2_/ether was identical with this synthetic compound on TLC, mp, and mmp.

### 3.5. 1-(2, 4, 5-Trimethoxy phenyl)-1-nitropropene (**2**)

A mixture of *β*-asarone (10 g, 48 mmol) in 150 mL of ethyl acetate containing iodine (18.28 g, 72 mmol) at 0°C was added to a solution of sodium nitrite (13.24 g, 192 mmol), ethylene glycol (8.93 g, 144 mmol), and water 20 mL. The reaction mixture was stirred at room temperature for 48 hrs under nitrogen and the ethyl acetate layer was separated, washed with water and then with 10% thiosulphate, and dried over MgSO_4_. Ethyl acetate layer was evaporated and recrystallized from methanol to obtain a yellow crystalline compound (70–75% yield).

MP: 158–160°C.

IR (KBr): 2948, 1645, 1346, 1214, 1032, 829, 769 cm^−1^.

^1^H NMR (CDCl_3_, 400 MHz): *δ* 1.80 (3H, d, *J* = 7.6 Hz, CH_3_), 3.76 (3H, s, OCH_3_), 3.83 (3H, s, OCH_3_), 3.93 (3H, s, OCH_3_), 6.57 (1H, s, H-3), 6.67 (1H, s, H-6), 7.40 (1H, q, *J* = 14.6 and 7.6 Hz, H-8).

^13^C NMR (CDCl_3_, 100 MHz): *δ* 152.64, 151.44, 149.58, 142.86, 133.74, 115.01, 109.64, 97.28, 56.73 (OCH_3_), 56.33 (OCH_3_), 56.05 (OCH_3_), 14.28 (CH_3_).

GC-MS (*m*/*z*) = 253 [M^+.^] (74), 207 (100), 192 (40), 177 (55), 161 (26), 149 (24), 131 (12), 121 (32).

### 3.6. 1-(2, 4, 5-Trimethoxy)phenyl-1-propanone (**3**)

To a solution of compound** 2** (1 g, 4.46 mmol) in methanol (25 mL) was added sodium borohydride (0.5 g, 3.2 mmol) and stirred the reaction mixture at 25–30°C for 30 minutes. Completion of the reaction was confirmed by TLC. The reaction mixture was concentrated under vacuum and acidified with dilHCl to pH about 4.0 and extracted with CH_2_Cl_2_ (2 × 15 mL) washed with water and dried over Na_2_SO_4_. Evaporation of CH_2_Cl_2_ layer followed by crystallization from methanol gave colourless crystals.

MP: 108-109°C.

IR (KBr): 2959, 1712, 1649, 1618, 1510, 1215, 1028, 810, 750 cm^−1^.

^1^H NMR (CDCl_3_, 200 MHz): *δ* 1.18 (3H, t, *J* = 7.2 Hz, CH_3_), 2.99 (2H, q, *J* = 14.5 & 7.2 Hz, CH_2_), 3.85 (3H, s, OCH_3_), 3.87 (3H, s, OCH_3_), 3.91 (3H, s, OCH_3_), 6.50 (1H, s, H-3), 7.43 (1H, s, H-6).

^13^C NMR (CDCl_3_, 50 MHz): *δ* 200.73 (C=O), 155.16, 153.58, 143.09, 119.19, 112.80, 96.56, 56.28 (OCH_3_), 56.18 (OCH_3_), 56.10 (OCH_3_), 37.07 (CH_2_), 8.63 (CH_3_).

GC-MS (*m*/*z*) = 224 [M^+.^] (38), 195 (100), 180 (10), 165 (5), 151 (10), 137 (12), 122 (32), 109 (5).

## 4. Conclusions

*β*-Asarone (2, 4, 5-trimethoxy-(*Z*)-1-propenylbenzene) when subjected to nitration gave 1-(2, 4, 5-trimethoxy phenyl)-2-nitropropene (**1**) and 1-(2, 4, 5-trimethoxy phenyl)-1-nitropropene (**2**). Preparation of compound** 2** is reported here for the first time. The cytotoxic activities of these nitro derivatives were compared with *β*-asarone in five human cancer cell lines namely MCF-7, SW-982, HeLa, PC-3 and IMR-32 using MTT assay. Except in breast cancer line (MCF-7) compound** 2** exhibited five- to tenfold increase in activity compared to *β*-asarone and twofold increase over compound** 1**.

## Supplementary Material

Copies of ^1^H-NMR, 13C-NMR, 1D-NOESY and GC-MS spectra of 1-(2, 4, 5-trimethoxy phenyl)-2-nitropropene (**1**), 1-(2, 4, 5-trimethoxy phenyl)-1-nitropropene (**2**) and 1-(2, 4, 5-trimethoxy) phenyl-1-propanone (**3**) are provided with the supplementary material available online.

## Figures and Tables

**Table 1:** Cytotoxic activity for nitro derivatives of *β*-asarone by MTT assay.

IC_50 _values (*μ*M)^a^					
Compounds	HeLa^b^	MCF-7^c^	SW-982^d^	PC-3^e^	IMR-32^f^
**1**	39.24 ± 4.40	73.65 ± 3.26	42.65 ± 11.42	30.05 ± 6.13	47.14 ± 8.43
**2**	25.03 ± 4.32	74.28 ± 6.28	11.84 ± 3.23	25.44 ± 6.76	18.10 ± 2.14
*β*-Asarone	126.11 ± 6.82	>150	129.35 ± 8.12	>150	>150
Camptothecin	3.09 ± 0.34	27.34 ± 9.89	9.15 ± 0.36	3.42 ± 0.35	5.07 ± 1.32

^a^IC_50_: each set of data represents mean ± S.D from three different test results in triplicate and is expressed as the concentration of test compound which inhibits the cell growth by 50%.

^b^HeLa: human cervical cancer; ^c^MCF-7: human breast cancer; ^d^SW-982: human synovial sarcoma; ^e^PC-3: human prostate cancer; ^f^IMR-132: human neuroblastoma.
