# Supplementary material for: Nitro Derivatives of Naturally Occurring β-Asarone and Their Anticancer Activity
Source: Int J Med Chem. 2014 Oct 1;2014:835485. doi: 10.1155/2014/835485 (PMC4207380; doi:10.1155/2014/835485)

[1] H-NMR spectra of (2, 4, 5-trimethoxy-(*Z*)-1-propenylbenzene)

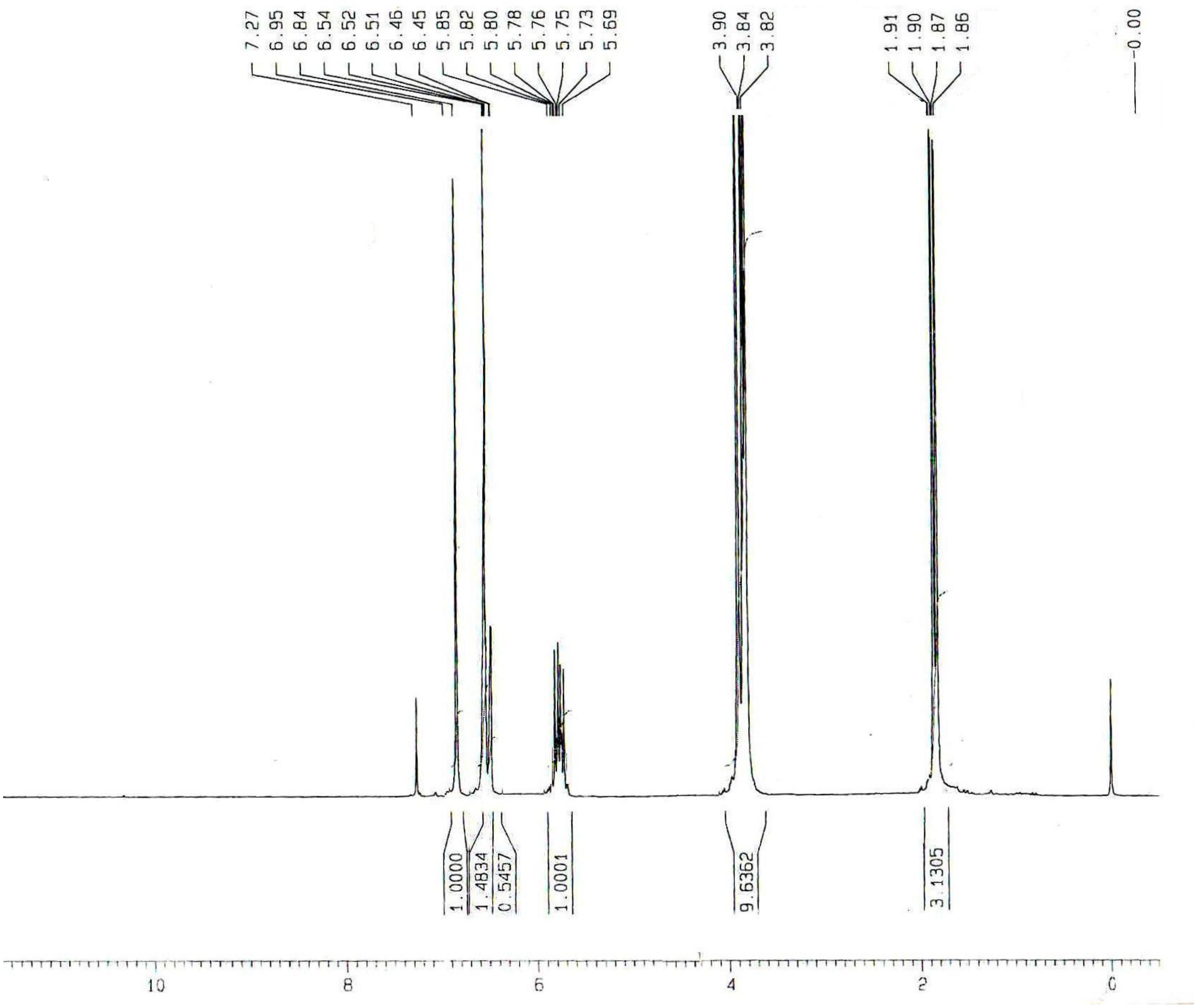

[13] C-NMR spectra of (2, 4, 5-trimethoxy-(*Z*)-1-propenylbenzene)

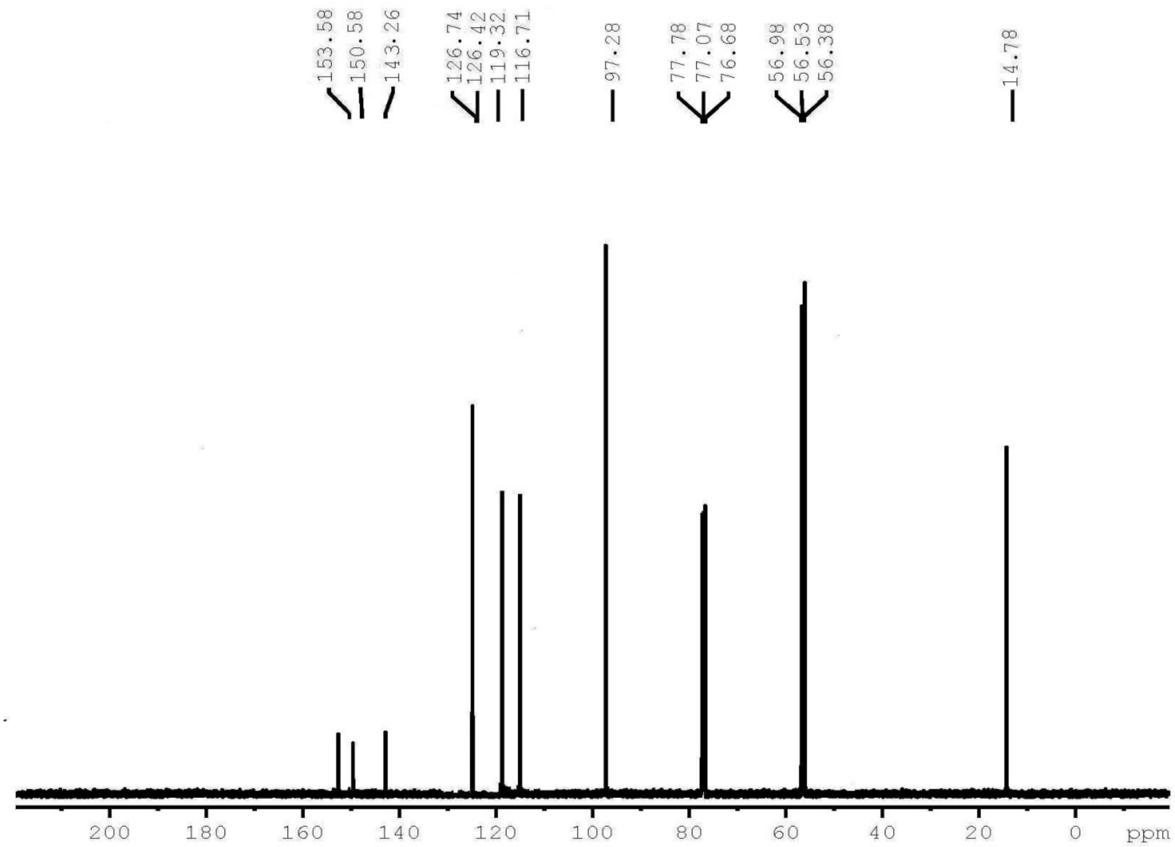

GC-Mass spectra of (2, 4, 5-trimethoxy-(*Z*)-1-propenylbenzene)

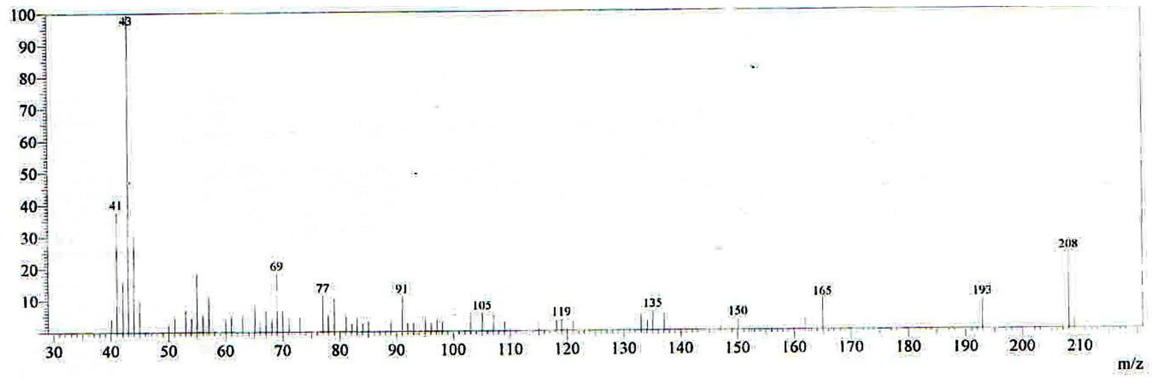

[1] H-NMR spectra of 1-(2, 4, 5-trimethoxy phenyl)-2-nitropropene (1).

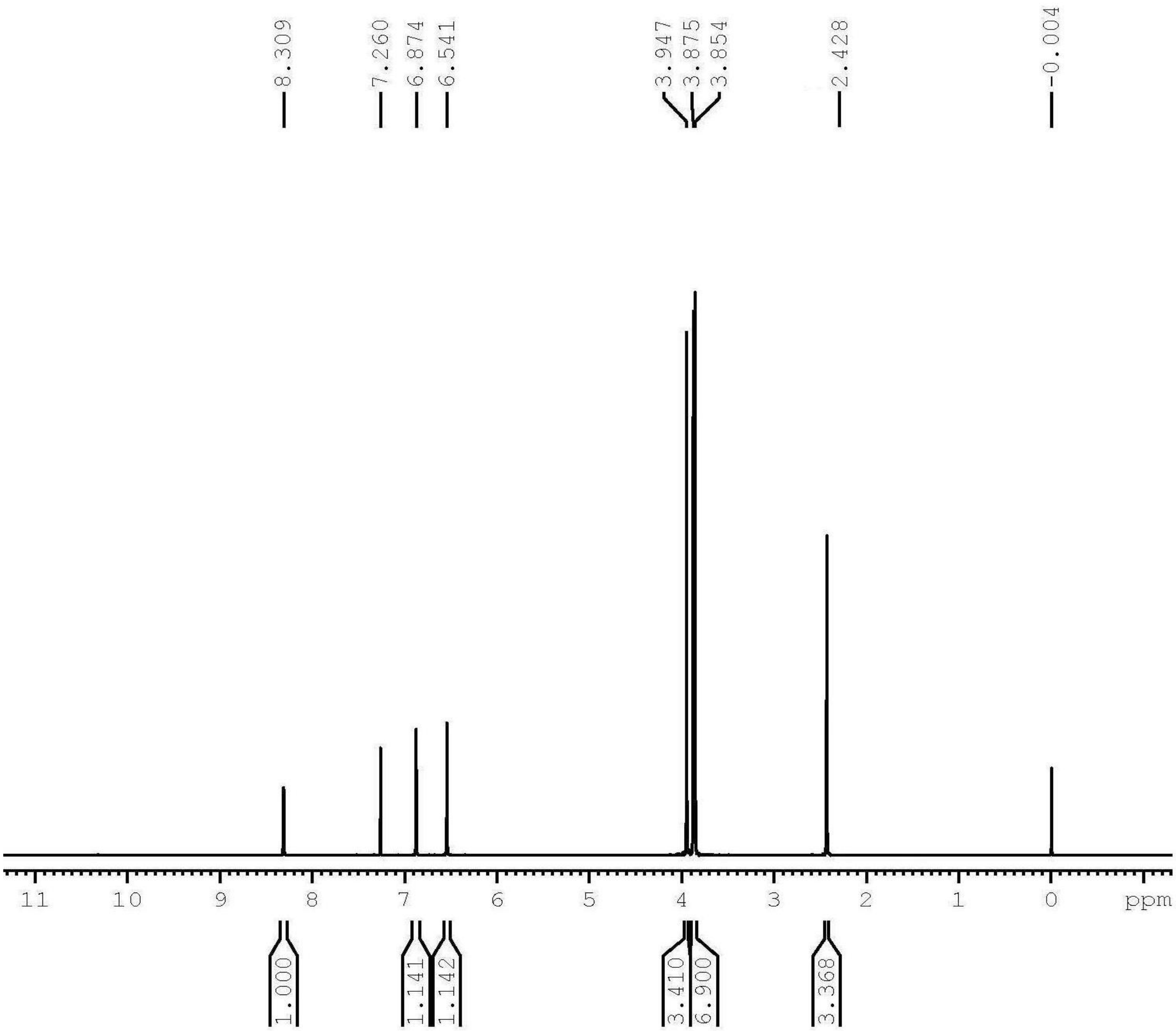

¹H-NMR spectra of 1-(2, 4, 5-trimethoxy phenyl)-2-nitropropene (1) with 1D NOESY experiment

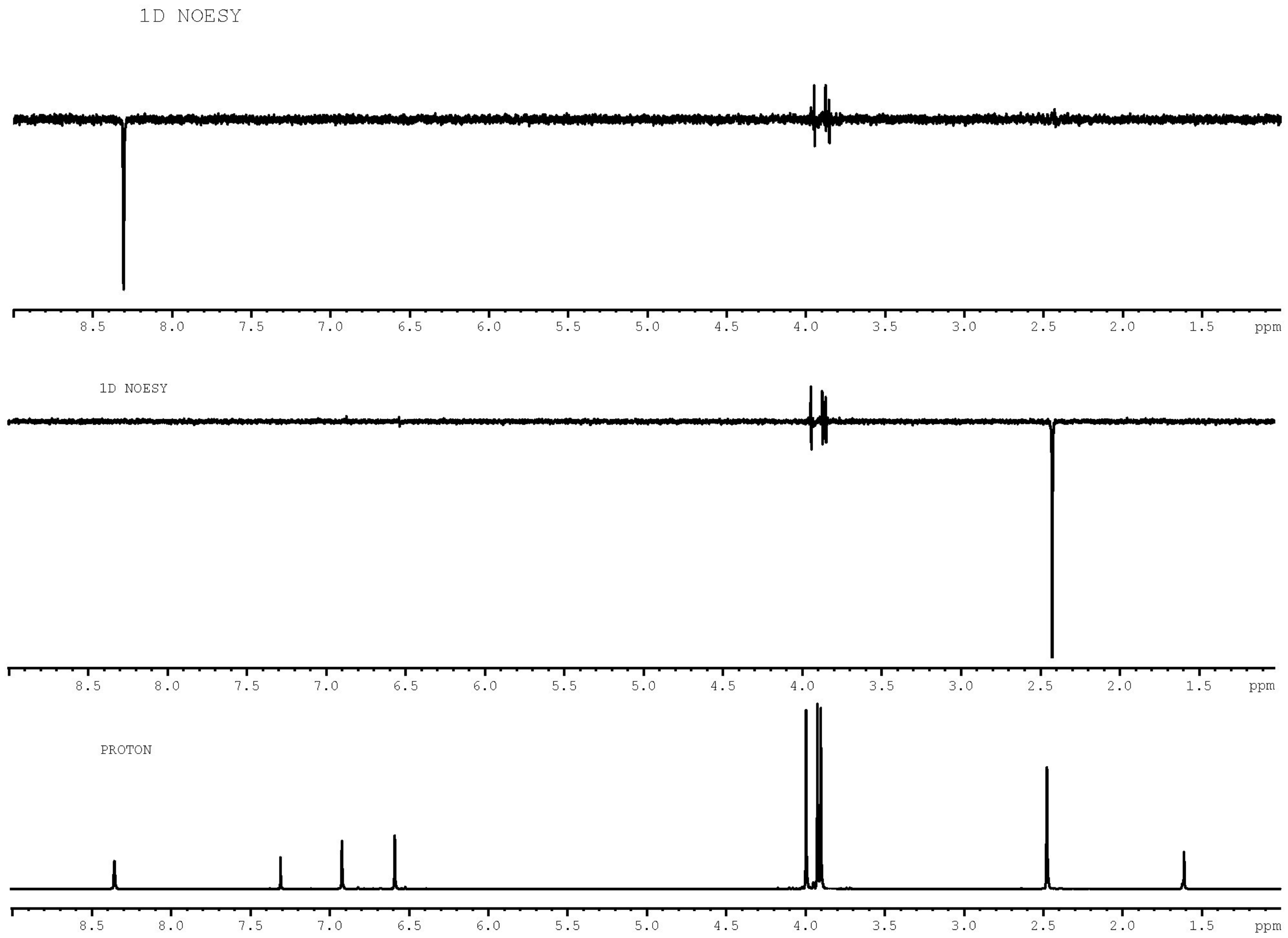

[13] C-NMR spectra of 1-(2, 4, 5-trimethoxy phenyl)-2-nitropropene (1).

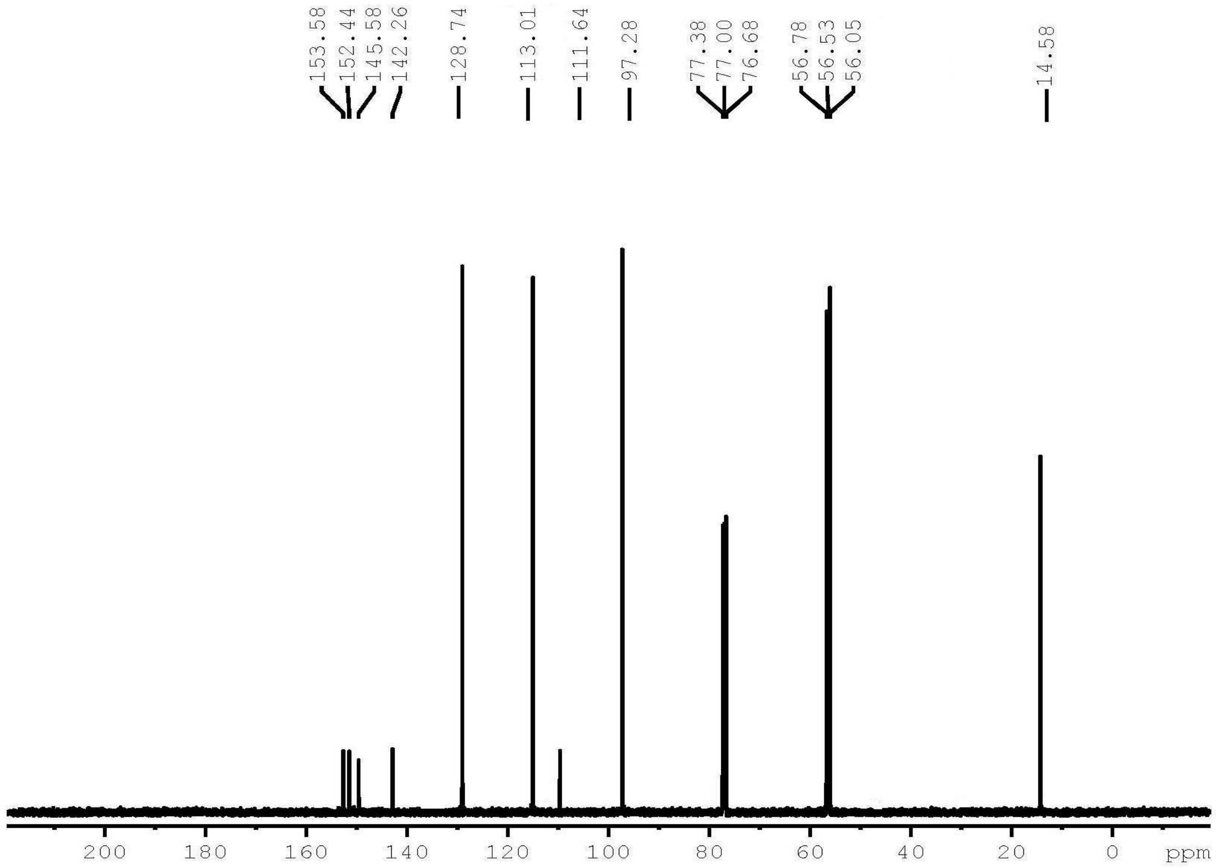

GC-Mass spectra of 1-(2, 4, 5-trimethoxy phenyl)-2-nitropropene (1).

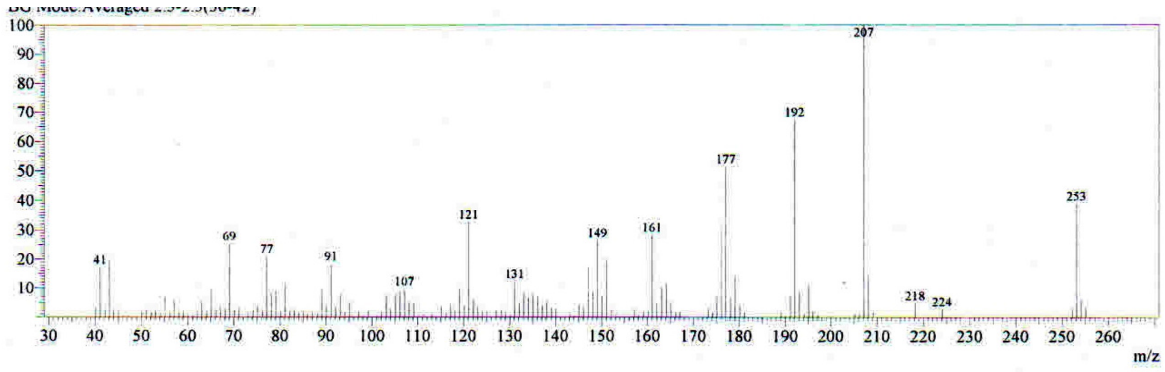

[1] H-NMR spectra of 1-(2, 4, 5-trimethoxy phenyl)-1-nitropropene (2)

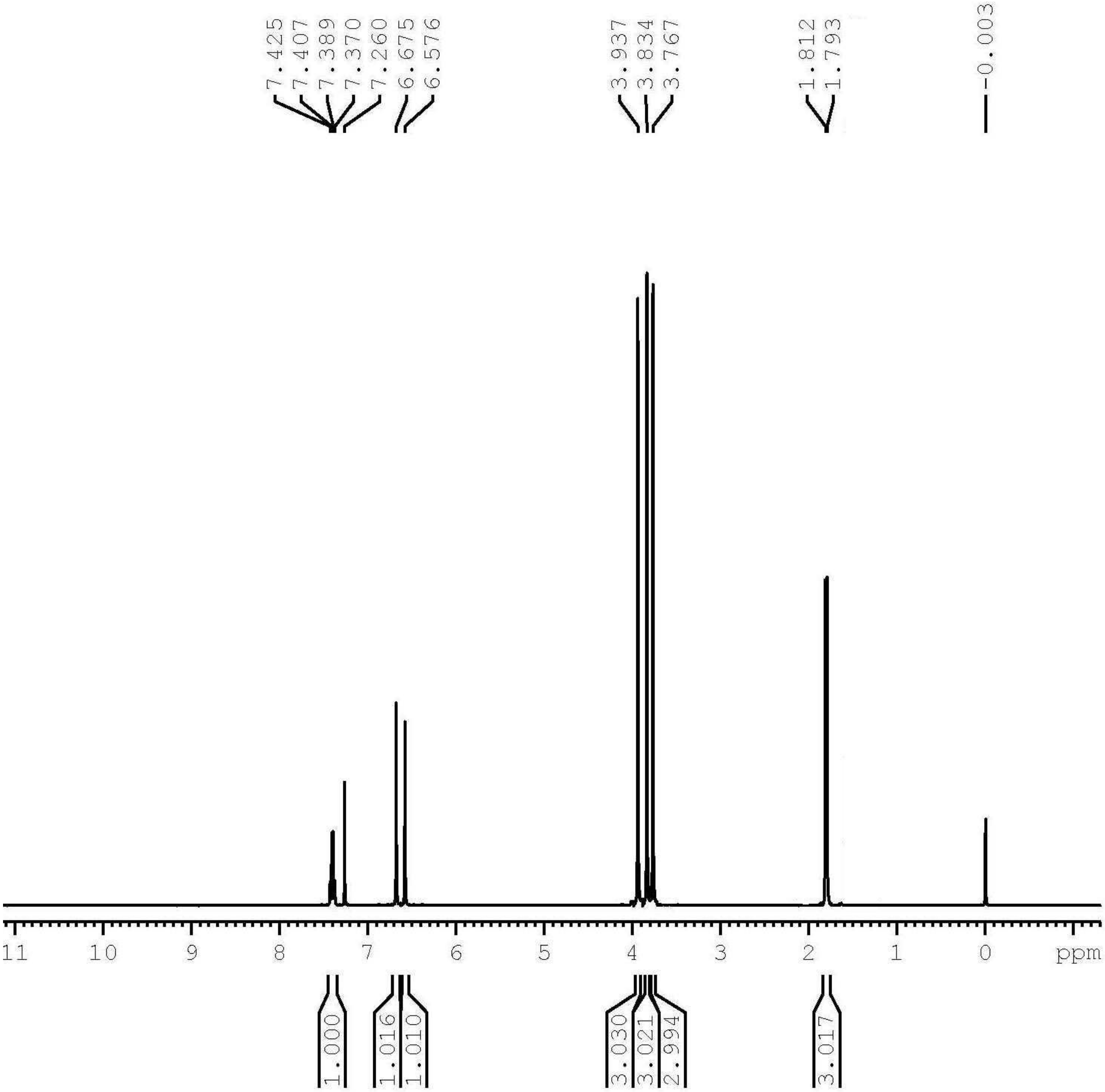

[1] H-NMR spectra of 1-(2, 4, 5-trimethoxy phenyl)-1-nitropropene (2) with 1D NOESY experiment

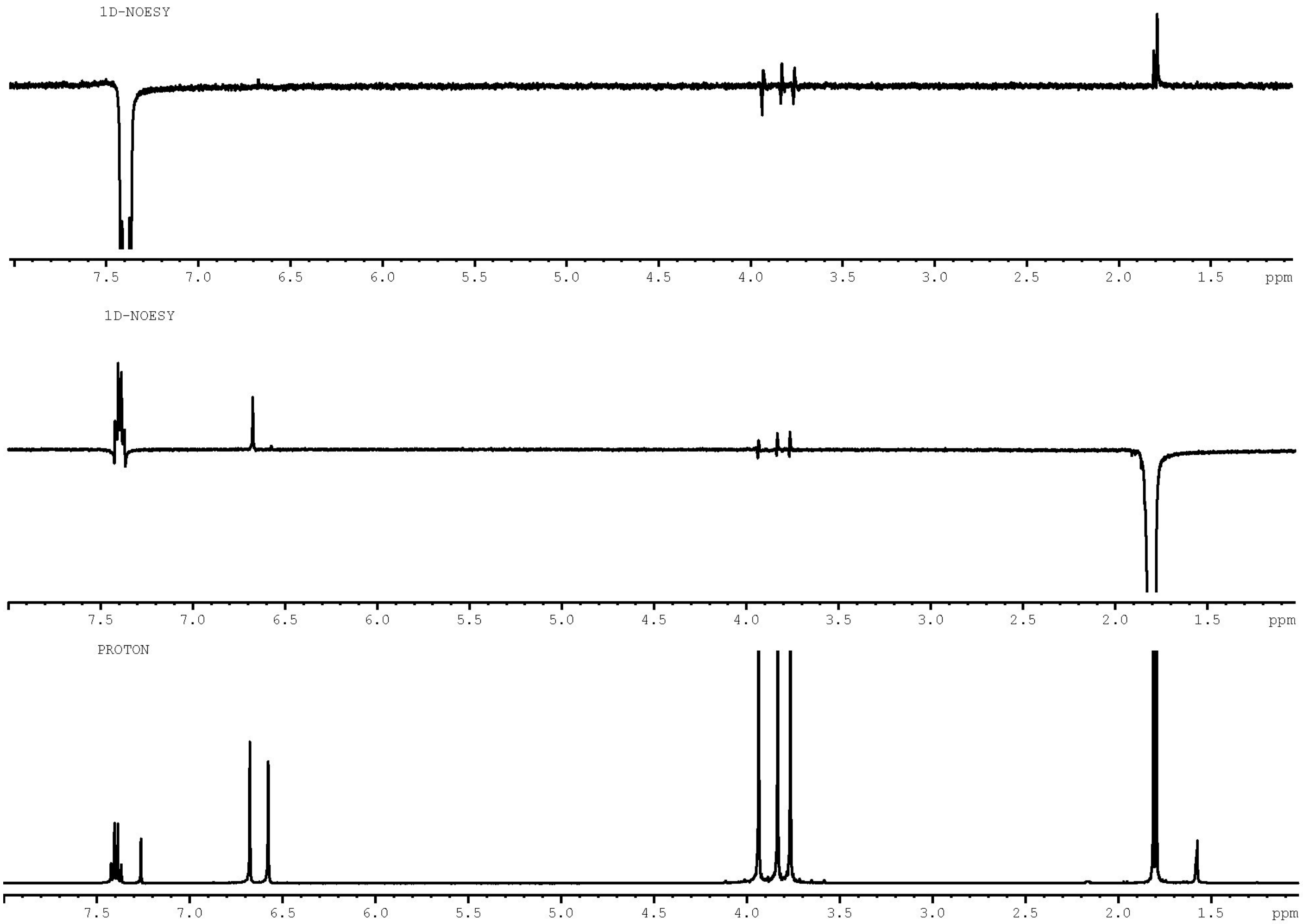

^{13}C-NMR spectra of 1-(2, 4, 5-trimethoxy phenyl)-1-nitropropene (2)

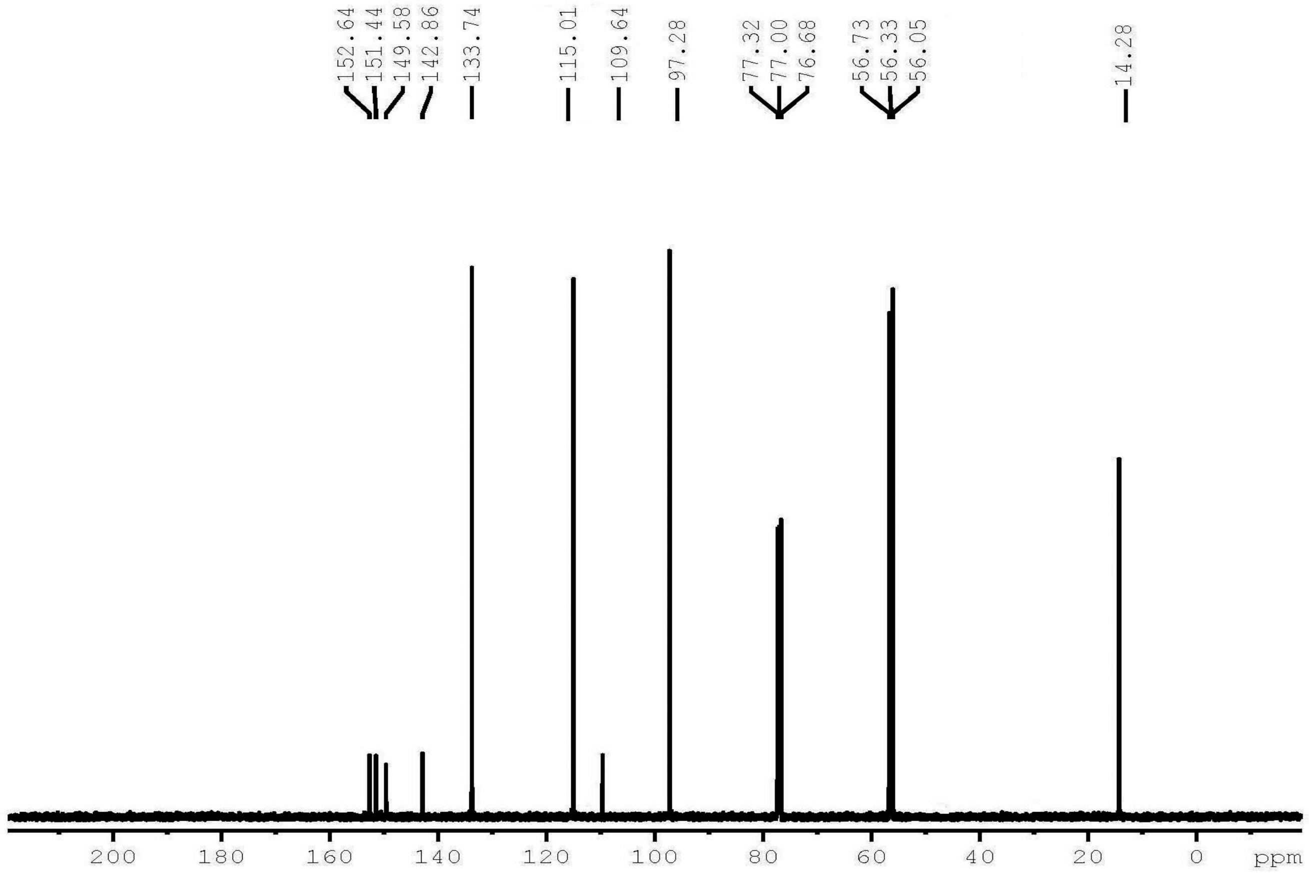

GC-Mass spectra of 1-(2, 4, 5-trimethoxy phenyl)-1-nitropropene(2)

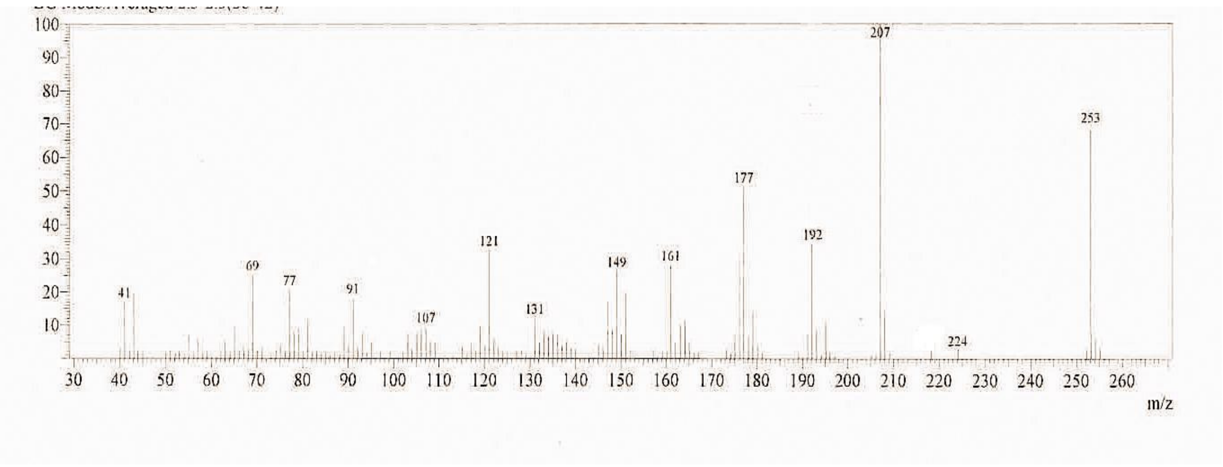

[1] H-NMR spectra of 1-(2, 4, 5-trimethoxy) phenyl-1-propanone (3)

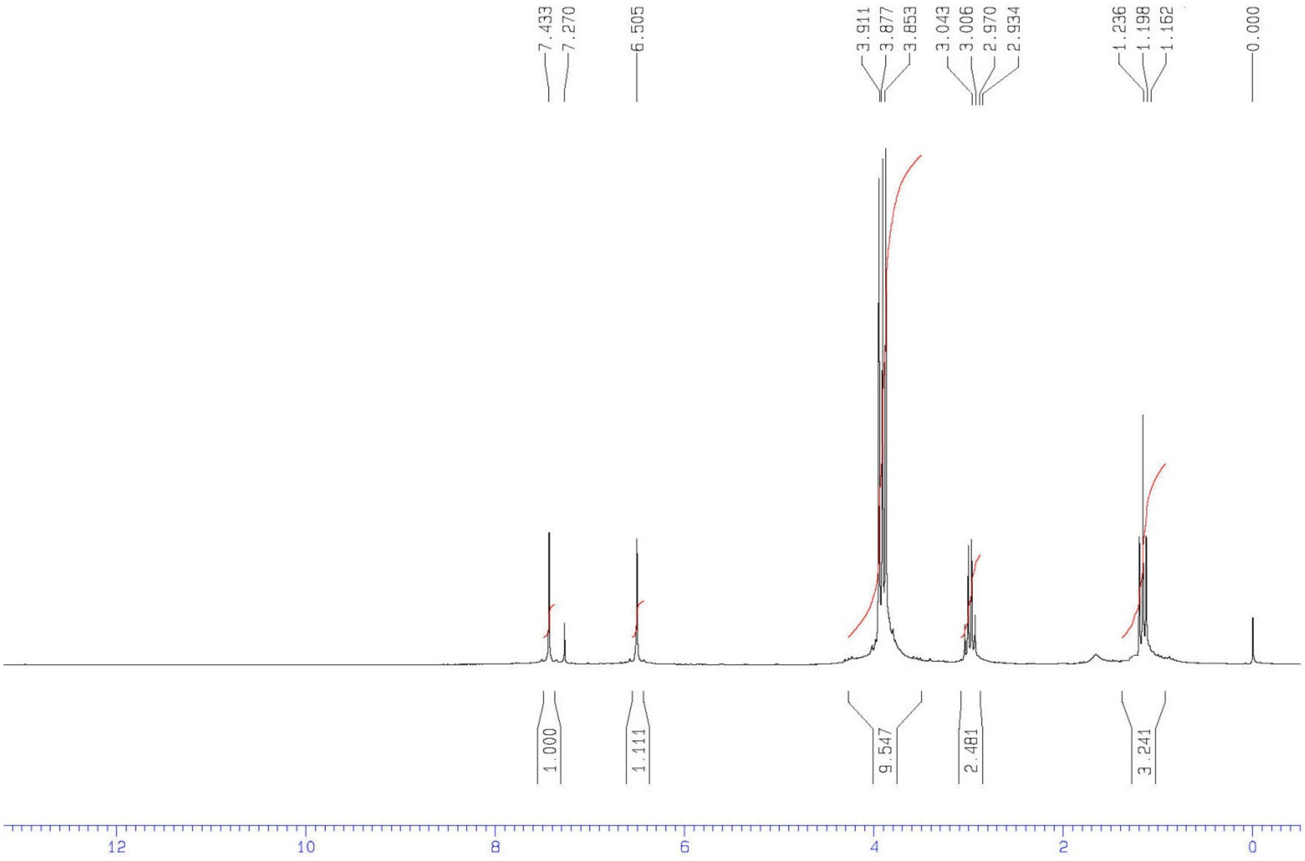

^{13}C-NMR spectra of 1-(2, 4, 5-trimethoxy) phenyl-1-propanone (3)

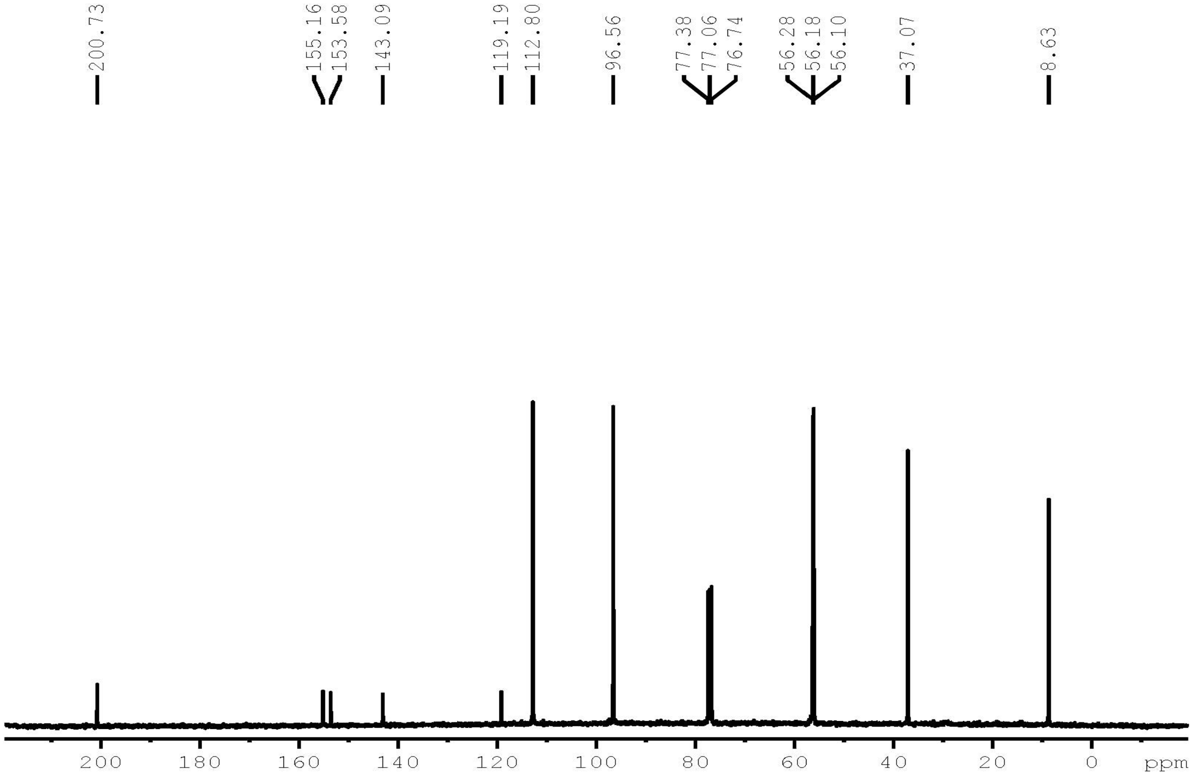

GC-Mass spectra of 1-(2, 4, 5-trimethoxy) phenyl-1-propanone (3)

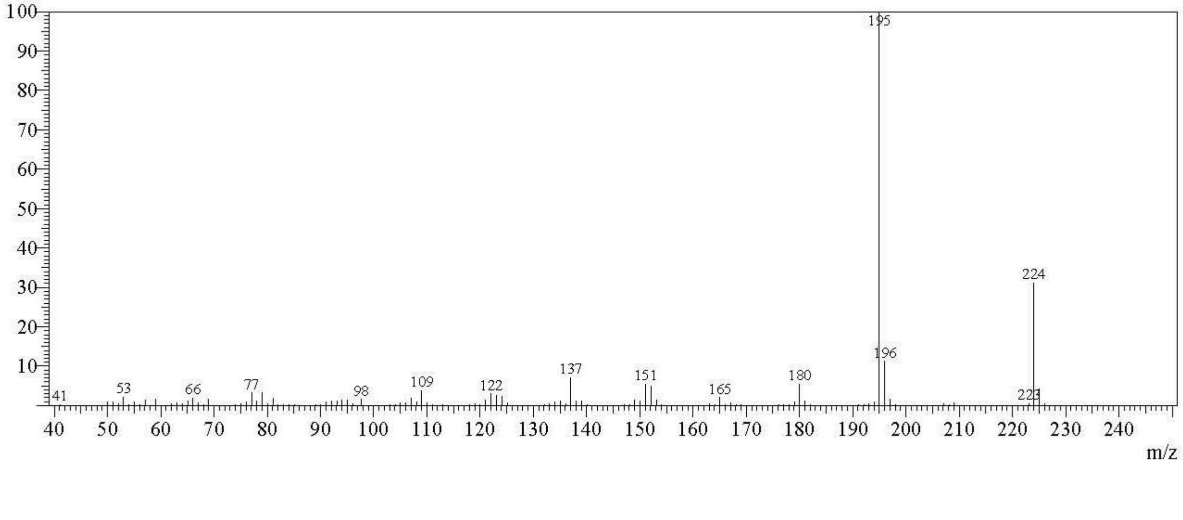

Supplement: Supplementary file 1 — Copies of 1H-NMR, 13C-NMR, 1D-NOESY and GC-MS spectra of 1-(2, 4, 5-trimethoxy phenyl)-2-nitropropene (1), 1-(2, 4, 5-trimethoxy phenyl)-1-nitropropene (2) and 1-(2, 4, 5-trimethoxy) phenyl-1-propanone (3) are provided with the supplementary material available online. [file 835485.f1.pdf]
